# Integrating Observation and Network Analysis to Identify Patterns of Use in the Public Space: A Gender Perspective

**DOI:** 10.3389/fpsyg.2022.898809

**Published:** 2022-06-02

**Authors:** Sergi Valera, Hernan Casakin

**Affiliations:** ^1^Department of Social Psychology and Quantitative Psychology, Faculty of Psychology, University of Barcelona, Barcelona, Spain; ^2^Social Environmental and Organizational Psychology Research Group (PsicoSAO), Barcelona, Spain; ^3^School of Architecture, Ariel University, Ariel, Israel

**Keywords:** public space, gender analysis, spatial patterns of use, systematic observation, network analysis, environmental psychology, urban design

## Abstract

In the last few decades, increasing attention has been given to gender issues in urban design. However, research on the urban environment continues to show large gender inequalities, which are especially evident when studying the use and enjoyment of the public space. This study aims to identify predominant patterns of use in public places and to explore the possible existence of traditional gender roles in the urban space. The study uses, three public spaces in the city of Barcelona as a case study, an innovative combination of systematic observation techniques and network analysis procedures. Variables collected by EXOdES, a dedicated software analysis tool for systematic observation, are represented as nodes of a network system and analyzed using network analysis tools. Findings confirmed that, in spite of the progressive consolidation of feminist urbanism, uses in the public realm resulting from traditional gender roles remained explicitly recognizable. Whereas women’s occupation of space was related to playground and resting areas, generally involving care activities concerned with children or elderly people, men were primarily located in resting and sports areas, practicing sports, or participating in leisure activities. These patterns of use were more prone to emerge when users were part of a group than when they were alone. From a gender perspective, a contribution of the study is that it informed about main aspects of the analyzed public spaces reconfirming the existence of traditional roles in society, and the significance of exploring the public space as a key scenario where social features are explicitly exposed. From a methodological perspective, the processing of observational data with network analysis tools proved to be relevant and suitable for dealing with the intricacies of urban place analysis. Compared to more classical approaches and systems, these techniques allowed to identify and interpret complex systems composed of many variables and relationships in a relatively straightforward manner, which turns it into a useful aid for urban designers and architects.

## Introduction

Nowadays, the interest in the public space has been renewed by actualizing the ideas of Lefebvre (1968) and Jacobs (1961), among others. Beyond the modern urbanism movement, the quality of urban life is, nowadays, drawn not only in terms of urban design and functionality but considering the social quality of city life. Indeed, public places are social places, where multiple interactions occur. As public space dynamics reflect what is socially relevant in a definite context (Andretta et al., 2015), the analysis of public urban places from a socio-environmental perspective leads to identifying social phenomena that affect the vitality of public life. Among these phenomena, the gender perspective analysis is one of the most relevant issues in the current urban debate. This study aims to identify predominant patterns of use in three public places in the city of Barcelona and to explore the possible existence of traditional gender roles in the urban space.

In traditional Western societies, women were assigned primary responsibility for childcare (Hartmann, 1981) and household activities (Schooler et al., 1984). These roles have dramatically affected their experience in public space, as well as their participation in leisure activities (Mowl and Towner, 1995). Franck and Paxson (1989) referred to several effects because of these. The most prominent one is that women’s mobility tends to be more restricted than that of men. The engagement in housekeeping and childcare led women to be less prone to benefit from the public space for discretionary use, often accompanied by little children or older people. In this regard, the presence of men is predominant in public spaces characterized by amenities such as betting, chess playing, or sports facilities such as baskets, football, and baseball courts. When alone, women tend to avoid public spaces perceived as unsafe, especially those with low maintenance or dirty. According to these scholars, compared with men, women are more likely to interact with others and are inclined to stay in populated spaces, particularly those inhabited by other women. Tackling these effects implies enhancing the publicness of public spaces, that is, increasing social diversity, variety of uses, and the acceptance of differences in terms of aspects such as gender, culture, ethnicity, and age. As stated by feminist urbanism, and the program for urban resilience from a gender perspective (Un-Habitat, 2018) urban design must rule over a key principle: if public spaces will be good for women, then they will also be good enough for everyone.

From the feminist urbanism perspective, the social role of high-quality urban places is crucial to promoting egalitarian use and enjoyment in the city. Urban life must reflect the diversity of existing social life, and the presence of disparate social groups -in terms of gender, race, origin, or status- because of their citizenship. In the words of Paravicini: “Urban living, particularly from the viewpoint of women, promises liberation from social control, from traditional gender roles and spatial designations” (Paravicini, 2003, p. 58). Accordingly, the urban role of high-quality public spaces can be regarded as of large importance since social, cultural, and gender-specific differences are perceived and experienced as an enhancement of urban living. However, it is fundamental to understand the extent to which these gender traditional roles are eradicated from the public realm. More research is needed to gain insight into the extent that which women are equally considered to men in what is called, following Lefevbre (1968), the right of the city. Much attention has been devoted to this issue in the feminist urbanism debate nowadays (Beebeejaun, 2017; see also www.right2city.org).

In spite of the increasing endorsement of the gender perspective in urban thinking and urban design (Little et al., 1988; McDowell, 1993; Bondi and Rose, 2003; Sánchez de Madariaga and Roberts, 2016), feminist urbanism studies show that with respect to urban settings, women’s needs and requirements are not considered as much as men. Recent research on urban analysis has shown large gender inequalities that involve mobility systems (Hanson, 2010; Pirra et al., 2021), urban facilities, and urban structure (Spain, 2014). To some extent, this situation occurs because cities are designed and managed mainly by men (Beebeejaun, 2017). Another reason is that urban space production is the outcome of social patriarchal structures, which impose barriers to women—both functional and symbolic—for the use and enjoyment of the urban public space (Blackstone, 2003; Bondi and Rose, 2003; Neaga, 2014).

Based on many key studies (Jacobs, 1961; Barker, 1968; Gehl, 1987, 2010; Barker, 2016; Sennett, 2017; Delclòs-Alió et al., 2019), public places can be conceptualized as complex systems. As such, they are made up of many variables (related to urban design, topological features, weather conditions, social interactions, individual dispositions, etc.) relating to each other in intricate configurations. It was Jane Jacobs who was among the first to view the public space as a place of complex and changing order. In the public space, people and groups—like dancers of a ballet—perform together in apparent chaos creating order within a hidden system. She referred to this aspect as “The art of the street” (Jacobs, 1961). This poetic, yet rigorous, account of street life offers a landmark example of what can be regarded as an ecological or systemic approach to space. This idea supposes considering human behavior as an outcome of socio-environmental system requirements. The assumption is that human behavior is a function of socio-environmental system demands. Among the many disciplines that have endorsed this approach are urban ecology and sociology. Consequently, in the words of Redman et al. (2004), an environment can be described as “a coherent system of (…) physical and social factors that regularly interact in a resilient, sustained manner, that is, a perpetually dynamic, complex system with continuous adaptation.” While understanding the public space as a complex network is a powerful concept, studies in environmental psychology literature that adopted methodological approaches from a systemic perspective are scarce. One of these is Roger Barker’s Behavior Setting Theory, which aims to investigate human behavior in daily contexts. According to Baker (2016), in a behavior setting, individuals, actions, and objects follow recognizable patterns. This theory sought to explain, perhaps for the first time scientifically, small-scale socio-environmental systems, as well as to study behavior in natural settings. However, as Popov and Chompalov (2012) highlighted, the behavior setting concept had very little impact on mainstream psychological theories (Wicker, 2002). In spite of this, Barker pioneered the ecological approach to psycho-environmental analysis, representing one of the most important perspectives in the discipline (Winkel et al., 2009).

Although the Behavior Setting Theory aimed at identifying environmental patterns of use, few studies have analyzed patterns involving social use of public places. Cao and Kang (2019) defined a pattern of use as “the ways that people use a space, which usually comprises activity and spatial occupancy” (op. cit., p. 189). The relationship between functionality and urban design was explored by Alexander (1977, 1979), who defined a pattern language as a way to analyze prototypical ill-structured urban design problems. He suggested a comprehensive list of standardized solutions to recurring urban uses and design situations that have influenced the way urban spaces are analyzed and designed today (Casakin, 2018). Gehl (1987) investigated environmental patterns and identified three types of human activities in urban places, including the necessary, optional, and social activities. For him, a public place ought to offer possibilities to develop all types of activities at different moments of the day, and for various users. This researcher proposed a set of valuable tools and a list of recommendations to observe urban social phenomena (Gehl and Svarre, 2013). Considering the process of privatization of public spaces and their progressive control surveillance, Cybriwsky (1999) studied the changing patterns of use of urban centers such as Battery Park City in New York and Yebisu Garden Place in Tokyo. These processes were found to restrict the social use of the space and reduce the quality of urban life. Goliènik and Thompson (2010) analyzed patterns of use in parks based on GIS and behavioral mapping. Cao and Kang (2019) studied four public places in the United Kingdom and China and using Hall’s (1966) social distance typology, they identified patterns of use that differ depending on if people were alone or in a group. In particular, women were more likely to stay in the group. Single users, on the other hand, were prone to be in the periphery, while groups were spatially distributed. Based on this literature, the current study depicts a pattern of use in the urban public space as a set of three interacting variables dealing with a user’s profile, a specific physical environment or space, and certain use of the space (Casakin and Valera, 2020; Valera, 2020).

What is common to the studies that analyze patterns of use is the systematic observation as a methodological approach (Cybriwsky, 1999; Goliènik and Thompson, 2010; Koen and Durrheim, 2010; Gehl and Svarre, 2013; Metha, 2014; Cao and Kang, 2019; Park, 2020)? Such an approach showed to be effective in the analysis of natural contexts (Anguera, 2003; Anguera et al., 2019) it is widely used in many recent studies. Differentiating itself from other empirical approaches such as self-report, systematic observation is a direct method that offers the possibility of collecting objective information with strong internal validity, while it allows the concurrent generation of data about the physical and social environment where an activity occurs (McKenzie and van der Mars, 2015; Park, 2020). This is, for instance, the methodology adopted by Iqbal and Ceccato (2016) to study the safety of urban parks in Stockholm considering CPTED criteria, and by O Caughy et al. (2001) to assess the impact of parks in urban neighborhoods’ on the well-being of families and children.

Finally, in previous studies, we dealt with the issue of gender analysis in public places by using an observational methodology (Pérez-Tejera et al., 2011, 2018). By analyzing a wide sample of public places, results offered much evidence on gender uses of the space (i.e., gender occupancy, preeminent activities, race profile, etc.). However, it was difficult to identify with precision, specific patterns of use that clearly related to gender roles. This issue is mainly because of the use of Polar Coordinates Analysis for exploring the collected data. This is a reduction data technique based on multievent sequential analysis (Bakeman and Quera, 2011) for exploring relationships between a focal behavior and one or more conditional behaviors (Gorospe and Anguera, 2000). Significant relationships are depicted in several vector maps indicating the extent to which the conditional behaviors are excitatory or inhibitory of the focal behavior. Despite its strengths, the Polar Coordinates Analysis technique presents some limitations, mainly concerned with the need to generate as many vector maps as the number of values of the observed category (known as focal behaviors), and the rest of the observed categories (known as conditional behaviors). Consequently, numerous separate analyses must be carried out, each one revealing only a partial aspect of the pattern of use. Hence, to arrive at more comprehensive conclusions, hard post-integration analysis of the partial results is required. To deal with this problem, it is necessary to explore techniques that allow an integrative analysis of a large amount of observational data straightforwardly.

Bearing this research gap in mind, the goal of this study is to detect predominant patterns of use in public places, as well as to analyze the extent to which the existence of traditional gender roles can be still detected. For this purpose, the current study relies on the innovative combination of systematic observation techniques and network analysis procedures (Casakin and Valera, 2020; Valera, 2020). To achieve these goals, it will be necessary to establish: (1) what are the main user’s profiles in public places, (2) what type of activities they are involved in, and (3) where they are located.

## Materials and Methods

### Case Studies

**FIGURE 1:**
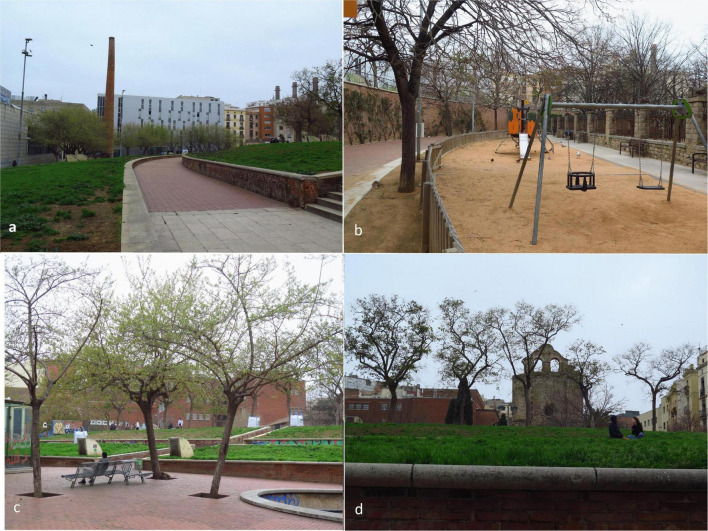
St. Pau del Camp Gardens, **(a)** Passage zone with the chimney of the old factory; **(b)** Playground area located in the lowest level of the park; **(c)** Resting area, located near the sport court; and **(d)** Artificial hills surrounding the Romanesque monastery of St. Paul in the Fields. Source: Authors.

The main reason why works dealing with the observation of urban spaces opt for the case study methodology (Cybriwsky, 1999; Metha, 2014; Cao and Kang, 2019; Park, 2020) is because it allows a focused evaluation of the object of study based on selected space attributes. In our study, three urban public places in the city of Barcelona were selected and analyzed with the aim of identifying patterns of use that disclose gender differences. In the last decades, Barcelona has become an example of urban design quality, characterized by a process of urban regeneration referred to as the “Barcelona Model” (Monclús, 2003; Doğan, 2021). While this model succeeded in the urban design realm, it was criticized for failing to produce significant social changes such as a more democratic use of the public space (Blakeley, 2005; Degen and García, 2012). This was particularly relevant for the public spaces located in low-middle class areas, such as the sites selected in the present study. While they can be categorized into different typologies of urban spaces (Carmona, 2010), the sites share several features that justify their inclusion in our study. Firstly, all three are public places created in the same historical period of the urban development of Barcelona. It was between the years 1980 and 2000, during the Barcelona Model period, when city planners acted in two major directions: conducting a large-scale urban renewal around the 1992 Olympic Games area (1992) and enforcing an urban design policy for new high-quality urban spaces, especially in areas previously neglected. Secondly, the selected case studies are located in traditional districts of the city such as Raval, Poble Sec, and Clot, characterized by historical deficiencies in urban facilities. And by a middle-low or low-class population. Finally, these places replaced totally or partially ancient factories, but at the same time, they conserve singular elements (e.g., chimneys, entrances, parts of walls, and big pieces of the original machinery) reminding the industrial past of an era. Consequently, we assume that the cases selected are appropriate for this study. These are described as follows:

St. Pau del Camp Gardens (Figure 1) is a 9,600 sq. m area created in 1992 in the Raval district to replace decaying buildings and an old factory, whose chimney remains in place. A large green area forms a small artificial hill to overcome the unevenness of the terrain caused by an underground parking lot. A series of gardens surround the Romanesque monastery of St. Paul in the Fields. Between the parterres (i.e., ornamental arrangement of flower beds), excavated semicircular paths extend along the medium-height walls designed to relax and enjoy. A sports court and a little playground complete the landscape.

**FIGURE 2:**
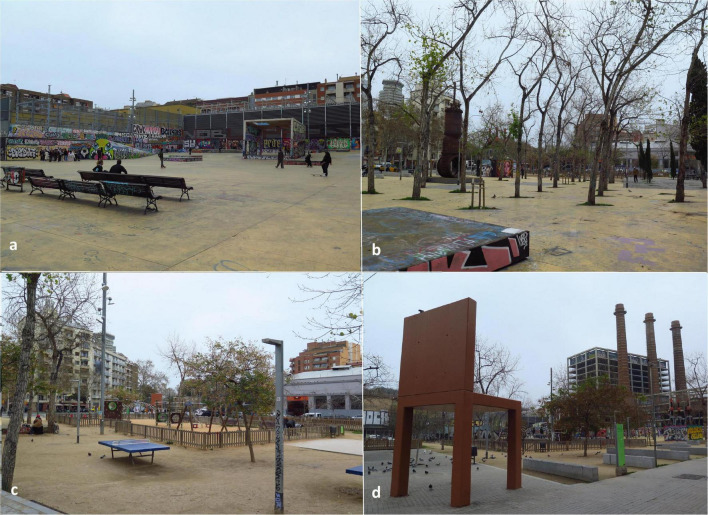
Three Chimneys Park, **(a)** The esplanade; **(b)** Passage area characterized by trees and parts of the old machinery used as sculptures; **(c)** Playground area, ping-pong tables, and resting zone with benches; and **(d)** Panoramic view of the iconic three chimneys that give the park its name. Source: Authors.

The Three Chimneys Park is in the Poble Sec district. Created in 1995, its name is an allegory to three big chimneys conserved from the original power station in Barcelona, popularly known as “The Canadian.” At the beginning of the 20th century, the company was a national pioneer in the production of electrical power. With an area of 8,890 sq.m, the park is structured into two main zones: one is a large central esplanade partly surrounded by a building and a low wall; it contains sitting places and a large concrete cube designed as a stage for celebrations. The other is a pavement area with geometrically organized tree plantations and a playground court containing ping pong tables (Figure 2).

**FIGURE 3:**
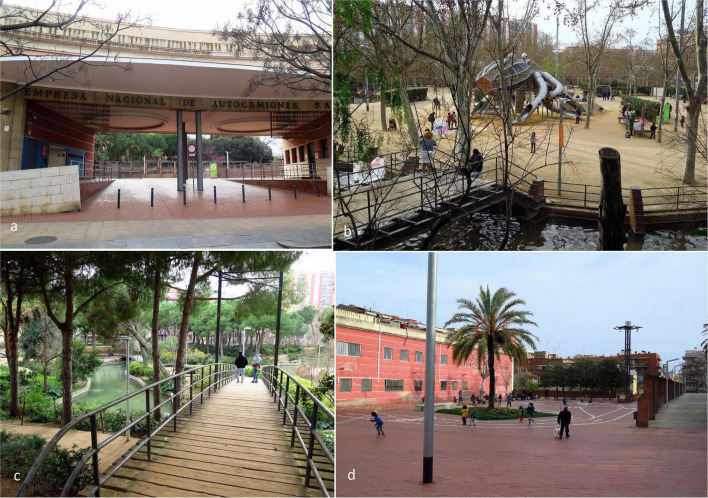
Pegaso Park, **(a)** Main entrance of the park, preserving the original access to the factory; **(b)** The lake surrounding the playground area in the esplanade; **(c)** Bridges, paths, and canal flowing to the lake; and **(d)** Paved playing area serving as a transition between the main entrance and the green areas. Source: Authors.

Like the other sites, Pegaso Park is a green area formerly occupied by an emblematic truck factory, located between the old historic districts of Sant Andreu and La Sagrera (Figure 3). Inaugurated in 1986, its name comes from the popular Pegaso trucks produced in this factory. With an area of 36,500 square meters, Pegaso Park is the largest of the three. The main entrance preserves part of the façade of the factory. The Park has organized into two main sectors: a shaded esplanade, bordered by Privet trees, a lake, and low hills. At the top of the park is a promenade surrounding a sports center and play areas for children connected to a lake. A water channel flowing from the lake is the major landscape element. It crosses the park diagonally running through exuberant and shady vegetation reminiscent of Riparian forests. Bridges as original as those inspired by the traditional Japanese architecture cross the canal at key points along its route.

### Observational Procedure

In this study, we used an N/F/M observational design (Anguera et al., 2001). N is for nomothetic (making observations of dissimilar places and groups of people), F is for intersessional follow-up (recording of several sessions), and M is for multidimensional (analysis of the multiple macro-criteria involved in the observational device).

With the help of the City Council of Barcelona, an observation tool (EXOdES) was created to explore how parks are used, and what are the main environmental features and activities that occur in space. The EXOdES instrument allows the recording of simultaneous behaviors involving multiple criteria through the combined use of category systems and field formats. In particular, it enables the classification of the data based on 26 dimensions organized under four macro-criteria: (i) context, (ii) groups and individuals’ profiles, (iii) activities, and (iv) environment. Information about the setting was coded according to its function (i.e., passage zone, resting zone, playground, green area, etc.). The observed individuals were coded according to gender (i.e., male, female), group age composition (i.e., child, teen, adult, elder), and ethnicity (i.e., White, Latin, Arab, Asian, and African). Groups of people were coded considering their size (i.e., 2, 3–5, 6–10, and 10–20), gender (i.e., men, women, mostly men, mostly women, and equally mixed), age composition (i.e., children, teens, adults, elders, children with teens, adults with elders, and children or teens with adults or elders), and ethnicity. Both individuals and groups were also coded regarding the presence or absence of signs of homelessness. Public space use was coded according to three indicators: main activity (i.e., talking or resting in place, picnicking, sleeping, playing, walking, practicing sports, and mental activities such as reading a book or playing cards), use of transport vehicles (i.e., motorized vehicles, bicycles, strollers, wheelchairs, and skates or roller skates), as well as the presence or absence of social incivilities (i.e., drunkenness, drug consumption or drug dealing, prostitution, urinating in public, and illegal street vending). Finally, the environmental assessment included aspects such as level of cleanliness, graffiti presence, level of maintenance of green areas, and visual control (Pérez-Tejera et al., 2011, 2018; Valera et al., 2018). Depending on the type of variables, systems of categories were created for criteria with a limited range of options (e.g., age, gender, and racial or ethnic group), and catalogs (field formats) were generated for criteria with unbounded options (i.e., type of transportation vehicles and type of activities) that could be expanded upon observing new responses. This up-to-date version of EXOdES included 280 mutually exclusive variables (codes).

**FIGURE 4:**
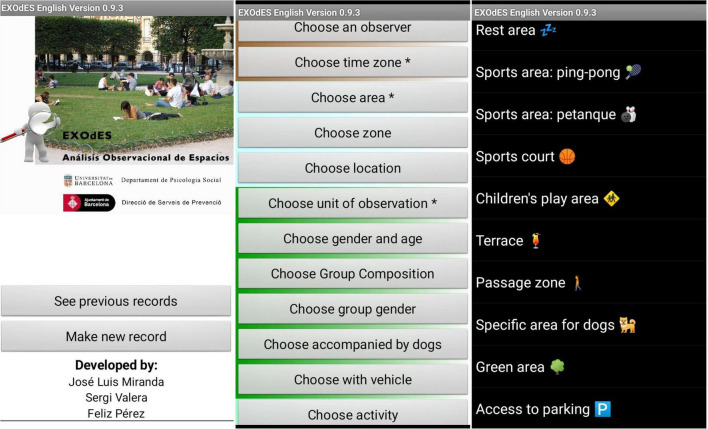
Screenshots illustrating the EXOdES tool.

Observations were carried out by six observers. The training was conducted by the researchers for a period of 4 weeks. It included practicing the coding process by using photographs and field-based observations until assuring high levels of inter-rater reliability. The quality of the data was controlled by the kappa Cohen’s coefficient, which exceeded 80%. To guarantee representativity of the use of spaces, all study sites were visited eight times per day (i.e., the observation period) during the following slots: 10:00–11:00, 11:00–12:00, 12:00–13:00, 13:00–14:00, 16:00–17:00, 17:00–18:00, 18:00–19:00, and 19:00–20:00. To reduce potential bias, at the end of the study each site was observed by at least three observers on three different weekdays. In a typical observation session, observers scanned the site and recorded all detected events. A scan is a visual sweep from left to right across the target area. Each record of EXOdES was represented synchronously as a set of variables (or dimension values) characterizing an event that occurs in a certain place. An EXOdES record included information about the observer, the place being observed, the date and the observational period, the demographic characteristics of an individual or group using the site, the activity being performed, and the environmental characteristics of where the activity takes place. In each location, events were observed for 45-min. They were recorded by means of an application for electronic devices (Figure 4) that allows using of EXOdES in an easy and discreet manner, avoiding a contamination effect from the observers. EXOdES application is available at www.exodes.org.

The final database comprised 3.122 records: 782 in St. Pau del Camp Gardens, 1.100 in Pegaso Park, and 1.240 in Three Chimneys Park. These records were collected during 108 sessions along different time slots (36 sessions per case study) representing 27 h per site, which is 81 h in total (see Table 1).

**TABLE 1:** Distribution of the hours of observation per case study.

	Case studies				
	
Observational period	ST.PAU DEL CAMP GARDENS	THREE CHIMNEYS PARK	PEGASO PARK	*n*	*M*
10:00 to 11:00	3	3	3	9	3
11:00 to 12:00	3,75	3	3,75	10,5	3,5
12:00 to 13:00	2,25	3,75	3	9	3
13:00 to 14:00	3	2,25	3,75	9	3
16:00 to 17:00	3	4,5	3,75	11,25	3,75
17:00 to 18:00	3,75	3	3	9,75	3,25
18:00 to 19:00	3,75	3,75	3,75	11,25	3,75
19:00 to 20:00	4,5	3,75	3	11,25	3,75
	27	27	27	81	

### Network Analysis

After collection, data were analyzed as a network system. Based on Graph Theory, studies on Network Analysis argued that a large part of the systems found in nature, even in society, can be described in terms of networks, which allows capturing the intricate web of connections between the units of which these networks are made (Wasserman and Faust, 1994; Cartwright and Harary, 1956; Palla et al., 2005; de Nooy et al., 2018). In our study, each record of EXOdES is considered a set of variables that are represented as nodes in a network and are related to each other in terms of co-occurrence. This principle is repeated throughout all observed events. As a result, a system of relationships between nodes is configured, generating non-directional networks that reveal how specific public space functions and behaves. To this aim, network analysis freeware software was employed. The collected observational information was analyzed using Pajek (de Nooy et al., 2018). This software represents data as networks through a combination of vertices and lines according to definite criteria. In our case, vertices (or nodes) were variables related to the selected observational categories, and lines were defined as co-occurrence relationships identified between these variables. Furthermore, Pajek provided indexes of both the network itself (centralization indexes) and the nodes involved (centrality indexes). Additionally, visualizations, clustering, and centrality indices provide critical information for understanding the dynamics of the network. Particularly, visualizations and clustering – i.e., grouping nodes by specific criteria – allow the representation of key components of the network. Once created, networks were exported from Pajek to VosViewer software (Van Eck and Waltman, 2011) via an available function of this application. This software was used for network visualization and clustering analysis. Options in normalization methods and clustering resolution allowed for obtaining optimal layouts for visualizing results.

This work was conducted in accordance with the Declaration of Helsinki. The study was exempt from ethics committee review and written informed consent, since: (a) it concerned the observation of people in public places where there was no expectation of privacy for those being observed; (b) The observer did not implement interventions or interactions with individuals or groups; and (c) No personal information captured by using photographs, films, or videos footage was shown in the research findings.

## Results

According to the definition of patterns of use adopted in the present study, several categories were selected for these results considering the proposed macro-criteria that included: (i) functional areas; (ii) gender profile of persons and groups; (iii) profile of groups (number of persons and age); (iv) activities; and (v) complimentary items such as dogs and vehicles. Categories of the environmental macro-criteria were excluded in the analysis due to their impossibility to discriminating between the potential patterns of use. In this regard, after testing them in preliminary network analyses, the regularity of variables related to these categories collected from different observation sessions did not allow to relate definite environmental features to identifiable patterns of use.

Finally, data were processed to obtain three networks, that is one per case study. Table 2 presents the main parameters and corresponding values for each of these networks. Three Chimneys Park is the biggest network, both in number of Vertices (nodes) and Lines (links between nodes). It also has the highest average Degree (average of connected nodes). However, St Pau del Camp has the highest Degree of Centralization, that is, the most compacted network. The density between networks is similar in all cases.

**TABLE 2:** Descriptive parameters of the networks obtained in each case study.

	ST. PAU DEL CAMP GARDENS	THREE CHIMNEYS PARK	PEGASO PARK
Number of vertices (*n*)	71	85	81
Total number of lines	481	726	634
Density	0.19356137	0.20336134	0.19567901
Average degree	13.54929577	17.08235294	15.65432099
Network degree centralization	0.57991718	0.53542742	0.49145570

While centralization indexes refer to the network as a whole, centrality indexes describe nodes that are involved in a specific network. Among these, Degree (*d*) and Weighted Degree (*Wd*) are crucial. The former refers to the number of nodes that are directly connected to a definite one. Its centrality derives from how many nodes appear together with it. Nevertheless, one node can be connected to another several times. The Weighted Degree index informs the number of connections (lines) received by a node: Its centrality derives from the number of times that this node appears in the observation sessions. Table 3 shows the main 40 nodes of each site’s network ordered by the Weighted Degree index and their respective Degree indexes.

**TABLE 3:** Weighted Degree (*Wd*) and Degree (*d*) of the 40 main nodes per case study network.

ST PAU DEL CAMP GARDENS			THREE CHIMNEYS PARK			PEGASO PARK		
				
Label	*Wd*	*d*	Label	*Wd*	*d*	Label	*Wd*	*d*
*Resting/Talking*	1,337	48	*Resting/Talking*	1,854	59	*Resting/Talking*	1,071	50
*Resting_area*	1,068	43	*Resting_area*	1,480	59	*Walking*	920	36
*Group_men*	682	53	*Group_men*	1,307	61	*Passage_area*	911	48
*Man*	470	20	*Esplanade*	682	47	*Resting_area*	874	49
*2_adults*	423	26	*Passage_area*	546	49	*Dog*	848	32
*Passage_area*	387	32	*Man*	540	31	*Group_men*	657	53
*Dog*	385	27	*Skate*	506	31	*Playing*	512	39
*Group_mixt_gender*	358	36	*Sport_skate*	465	30	*Group_women*	493	43
*Walking*	308	30	*3to5_young*	420	27	*Playground*	490	30
*3to5_adults*	243	23	*2_young*	407	33	*Man*	486	26
*Group_women*	223	28	*Group_mixt_gender*	399	42	*Group_mixt_gender*	469	54
*Playground*	197	28	*Group_women*	368	40	*Woman*	317	18
*Green_area*	190	32	*Playground*	363	33	*2_child_adult*	315	19
*Playing*	187	64	*2_adults*	327	26	*Esplanade*	308	41
*Woman*	157	18	*Playing*	283	35	*Stroller*	278	26
*2_young*	144	15	*Walking*	226	28	*2_adults*	271	23
*2/more_dogs*	139	29	*Old_man*	223	16	*Old_man*	229	13
*Sleeping*	105	18	*Boy*	218	22	*3to5_child_adults*	161	18
*2_child_adult*	101	11	*2_child_adult*	188	20	*2/more_dogs*	161	33
*Esplanade*	95	22	*Dog*	181	26	*2_old*	123	15
*Boy*	83	18	*Stroller*	177	22	*3to5_adults*	113	20
*Old_man*	77	11	*Bike*	175	27	*Bike*	101	31
*3to5_child_adults*	75	12	*Woman*	165	21	*2_child*	99	12
*3to5_young*	73	14	*3to5_child_adults*	144	20	*Boy*	93	15
*Eating*	69	17	*Sculpture*	116	26	*Work_maintenance*	93	12
*Dogs_area*	63	15	*Sport_biking*	100	15	*Blue_space*	91	23
*Stroller*	61	20	*3to5_child*	96	18	*2_young*	89	16
*Sculpture*	58	11	*3to5_adults*	96	14	*Petanque_court*	82	22
*Work_maintenance*	52	12	*Sleeping*	83	13	*Reading*	81	14
*Bike*	50	15	*2_old*	82	12	*3to5_young*	71	15
*Automobile*	42	15	*Reading*	82	15	*Old_woman*	60	9
*Girl*	34	6	*Automobile*	78	18	*Green_area*	56	17
*5to10_adults*	34	8	*Parking_area*	76	20	*Girl*	55	10
*5to10_young*	33	13	*Scooter*	75	22	*Stairs*	52	18
*Stairs*	31	10	*Work_maintenance*	70	18	*Eating*	50	17
*Surveillance*	31	10	*2_child*	66	17	*5to10_child_adult*	48	8
*2_child*	24	9	*Eating*	63	18	*3to5_child*	47	16
*Child_male*	22	14	*Sport_football*	62	15	*3to5_old*	41	14

*Pink color represent activities; green color represents functional areas; blue color represents gender profiles; yellow color represents groups profiles; and gray color represents complementary items such as dogs and vehicles.*

The main activity carried out in the observed places is *Resting* (when people are alone) or *Resting/Talking* (in the case of groups). In fact, this activity represents one of the most relevant nodes in the network. In all three case studies, it is the first ranked activity with respect to Weighted Degree, and the most important one regarding Degree measures. Together with *Passage areas* related to *Walking* activity, resting areas (*Resting_area*) are the largest visited places, except in Three Chimneys Park where *Esplanade* is also relevant. Furthermore, in general, people appear in the public space in groups rather than individually. The presence of males, especially groups of men (*Group*_*men*), is dominant in the places studied. In contrast, the presence of women is less frequent, and when it happens, they appear in group (*Group*_wo*men*). This effect is more relevant in St. Pau del Camp Gardens (*Wd_*Group_men*_* = 682; *d_*Group_men*_* = 53; *Wd_*Man*_* = 470; *d_*Man*_* = 20 in front of *Wd_*Group_women*_* = 223; *d_*Group_women*_* = 28; *Wd_*Woman*_* = 157; *d_*Woman*_* = 18) and Three Chimneys Park (*Wd_*Group_men*_* = 1307; *d_*Group_men*_* = 61; *Wd_*Man*_* = 540; *d_*Man*_* = 31 in front of *Wd_*Group_women*_* = 368; *d_*Group_women*_* = 40; *Wd_*Woman*_* = 165; *d_*Woman*_* = 21) than in Pegaso Park (*Wd_*Group_men*_* = 657; *d_*Group_men*_* = 53; *Wd_*Man*_* = 486; *d_*Man*_* = 26 in front of *Wd_*Group_women*_* = 493; *d_*Group_women*_* = 43; *Wd_*Woman*_* = 317; *d_*Woman*_* = 18). The presence of dogs is prominent in St. Pau del Camp Gardens and Pegaso Park, while the skaters (*Skater*) and *Skating* activity are preeminent in the Three Chimneys Park.

As noted, a pair of nodes can be interconnected with each other several times. Depending on the number of connections, the value of the line (i.e., link) between two nodes can vary accordingly. The value of the lines that link gender profile options with both, functional areas and activities was calculated. Since these values are absolute, to normalize them in relation to the network we elaborated an index representing the percentage of the number of connections that link two nodes (value of the line) of the total number of connections of the network. The resulting indexes allowed comparison among the three case studies. Table 4 shows the index value between gender profile and functional areas, and Table 5 depicts the index value between gender profile and use of the space in each case study.

**TABLE 4:** Gender index by functional areas.

Case study (total network lines)					

ST.PAU DEL CAMP (481)		*Playground*	*Resting _area*	*Passage_area*	Leisure area (1)
	*Man*	0,416	25,571	9,355	6,029
	*Woman*	0,208	2,910	5,405	1,663
	*Child_male*	0,416	0,415	0,623	0,415
	*Child_female*	–	–	0,623	0,207
	*Boy*	0,208	3,950	1,039	1,455
	*Girl*	–	0,831	1,663	–
	*Old_man*	–	4,158	1,663	0,415
	*Old_woman*	–	–	–	–
	*Group_men*	2,079	27,442	4,158	6,237
	*Group_women*	7,692	3,950	1,247	1,663
	*Group_mixt*	2,287	13,097	2,702	3,534

**THREE CHIMNEYS PARK (726)**		***Playground***	***Resting _area***	***Passage_area***	**Leisure area (2)**

	*Man*	0,551	20,385	5,509	4,545
	*Woman*	0,964	3,168	2,066	2,617
	*Child_male*	0,275	0,413	0,275	0,964
	*Child_female*	0,413	–	-	0,137
	*Boy*	0,137	5,785	1,101	3,581
	*Girl*	0,137	1,790	0,550	0,551
	*Old_man*	–	11,157	2,066	0,551
	*Old_woman*	–	0,964	0,137	–
	*Group_men*	4,958	19,559	8,126	18,732
	*Group_women*	6,887	6,611	1,515	1,377
	*Group_mixt*	1,928	9,228	2,892	1,790

**PEGASO PARK (634)**		***Playground***	***Resting _area***	***Passage_area***	**Leisure area (3)**

	*Man*	–	10,725	13,880	5,362
	*Woman*	0,315	5,520	8,517	3,627
	*Child_male*	–	0,157	–	0,315
	*Child_female*	–	–	0,157	-
	*Boy*	0,315	1,892	2,366	0,788
	*Girl*	–	1,261	1,577	0,315
	*Old_man*	–	7,097	5,836	1,419
	*Old_woman*	0,157	1,262	1,412	0,630
	*Group_men*	7,255	8,832	5,047	8,517
	*Group_women*	10,094	5,520	5,520	2,208
	*Group_mixt*	5,205	7,571	4,889	4,258

*Average of lines linking areas with gender profile per case study.*

*(1) Esplanade, Sport_court, Petanque_court, Blue_space, and Green_area.*

*(2) Esplanade, Sport_court, Petanque_court, and Blue_space.*

*(3) Esplanade, Sport_court, Petanque_court, Blue_space, and Green_area.*

**TABLE 5:** Gender index by activity.

Case study (total network lines)						

ST.PAU (481)		*Resting*	*Walking*	*Playing*	Sport (1)	Working (4)
	*Man*	26,819	7,900	–	–	0,831
	*Woman*	7,277	3,950	–	–	0,208
	*Child_male*	0,624	0,415	0,415	–	–
	*Child_female*	0,208	0,623	–	–	–
	*Boy*	4,366	1,455	0,208	–	–
	*Girl*	0,416	1,663	–	–	–
	*Old_man*	5,198	1,455	–	–	–
	*Old_woman*	–	–	–	–	–
		***Talking***				
	*Group_men*	34,927	1,039	2,079	0,831	2,702
	*Group_women*	6,237	1,039	7,068	0,208	0,208
	*Group_mixt*	14,761	1,871	2,494	–	1,455

**THREE CHIMNEYS (726)**		***Resting***	***Walking***	***Playing***	**Sport (2)**	**Working (4)**

	*Man*	21,212	2,203	–	2,203	0,826
	*Woman*	4,821	2,066	0,275	0,275	1,652
	*Child_male*	0,275	–	0,275	1,239	–
	*Child_female*	–	–	0,413	0,137	–
	*Boy*	7,300	0,137	0,137	4,132	–
	*Girl*	1,652	0,275	–	0,688	–
	*Old_man*	10,606	1,652	–	0,137	–
	*Old_woman*	0,964	0,137	–	–	–
		***Talking***				
	*Group_men*	27,410	1,239	4,958	19,696	0,551
	*Group_women*	10,055	0,413	4,958	0,826	–
	*Group_mixt*	11,570	1,652	1,652	0,964	0,275

**PEGASO (634)**		***Resting***	***Walking***	***Playing***	**Sport (3)**	**Working (4)**

	*Man*	10,725	12,618	0,315	1,261	1,419
	*Woman*	7,413	9,621	0,157	–	0,157
	*Child_male*	0,157	–	–	0,157	–
	*Child_female*	–	–	–	–	–
	*Boy*	1,892	2,523	0,157	0,631	–
	*Girl*	0,631	2,050	–	0,157	–
	*Old_man*	5,993	6,151	–	–	–
	*Old_woman*	1,735	1,735	–	–	–
		***Talking***				
	*Group_men*	12,776	3,470	8,832	3,154	2,523
	*Group_women*	7,097	5,047	9,306	0,946	0,315
	*Group_mixt*	10,094	4,574	5,362	0,946	1,104

*Average of lines linking activities with gender profile per case study.*

*(1) Sport_football, Sport_basket, and Sport_biking.*

*(2) Sport_football, Sport_basket, Sport_skate, Sport_petanque, Running, Stretching, Sport_biking, and Sport_cricket.*

*(3) Sport_football, Sport_basket, Sport_petanque, Running, Stretching, Sport_bike, Sport_taichi, and Sport_kickboxing.*

*(4) Work_maintenance, and Surveillance.*

Analyzed by case studies, St. Pau del Camp Gardens’ functional areas are linked with different gender profiles. *Playground_area* relates to groups of women (*Group*_wo*men*), while resting (*Resting_area*) and leisure areas (*Esplanade, Sport_court, Petanque_court, Blue_space, and Green_area*) are connected to *Man* or groups of men (*Group*_*men*) (Table 4). *Playing* activities relate to groups of women (*Group*_wo*men*), whereas resting and talking activities (*Resting/Talking*) are linked to male users (*Man, Group_men*) (Table 5).

Similar results are observed in Three Chimneys Park. The playground area is linked to groups of women (*Group*_wo*men*), leisure areas (*Esplanade, Sport_court, Petanque_court*, and *Blue_space*) to groups of men (*Group*_*men*). Both *Resting_area* and *Passage_area* are largely related to male users (*Man, Group_men*) (Table 4). While there is no difference between men and women in *Playing* and *Walking* activities, the male gender is clearly linked to resting, talking (*Resting/Talking*), and sports activities (mainly *Sport_skate, Sport_biking*, and *Sport_football*) (Table 5).

As regards Pegaso Park, like, in the other sites, *Playground_area* is related to groups of women (*Group*_wo*men*), as well as resting (*Resting_area*) and leisure areas (*Esplanade, Sport_court, Petanque_court, Blue_space*, and *Green_area*) either composed of solitary men (*Man*) or groups of men (*Group*_*men*). Conversely, passage zones (*Passage_area*) are mainly connected to singles (mainly *Man* but also *Woman*) (Table 4). In addition, activities such as resting or talking (*Resting/Talking*) are predominant for male users (*Man, Group_men*), although no differences exist in *Playing* activities regarding gender (Table 5).

When the analysis of the main components of the networks is carried out integrally, it is possible to identify patterns of use of the sites based on the visualizations of the resulting networks. For a better visualization of the results, networks generated by Pajek were exported to VOSViewer. The network visualizations generated by VOSViewer allowed clearly capturing the global structure of the nodes and their relationships. In our case, the size of the nodes is represented according to the Weighted Degree index. Hence, the bigger the node, the larger the Weighted degree, and the larger the presence of this node on the network. The larger the central location of the node, the larger its relevance. Moreover, the closer one node is to another, the larger their interconnection in terms of co-presence. The size of the links between nodes are represented according to the value of the line (i.e., number of connections between pairs of nodes). Therefore, the thicker the line the higher the value of the line. Additionally, VOSViewer allows the application of cluster algorithms to the network, highlighting sets of nodes that repeatedly appear together. Depending on what information the sets or clusters contain, nodes related to users’ profile, activities, and functional areas, they can be related to specific as patterns of use.

**FIGURE 5:**
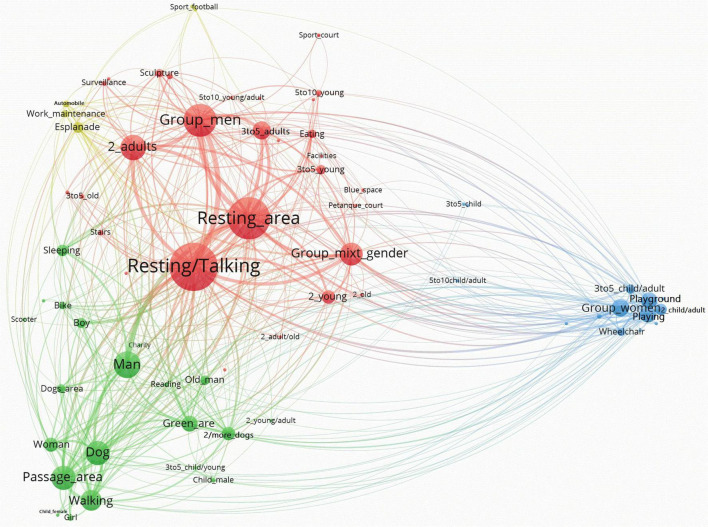
St. Pau del Camp Garden’s network by VOSviewer.

From a gender perspective, three main clusters define St. Pau del Camp Gardens’ network (Figure 5). The green cluster is defined by single persons of both genders (*Man*, *Woman*), accompanied by dogs (*Dog*, *2/more_dogs*), preferentially walking (*Walking*) in passage zones (*Passage_area*) or green areas (*Green_area*). The red cluster is related to groups of men (*Group*_*men*). This node is linked with the main nodes regarding Weighted Degree: talking and resting activities (*Resting/Talking*), and resting areas (*Resting_area*). Finally, the blue cluster outlines groups of women (*Group*_wo*men*) with children (*2_child, 3to5_child/adult*) and older people (*2_child_old*) (with presence of *Wheelchair*), involved in *Playing* activities are located in the *Playground* area. Interestingly, this cluster is well separated from the others, suggesting that the pattern of use is highly specific for this site.

**FIGURE 6:**
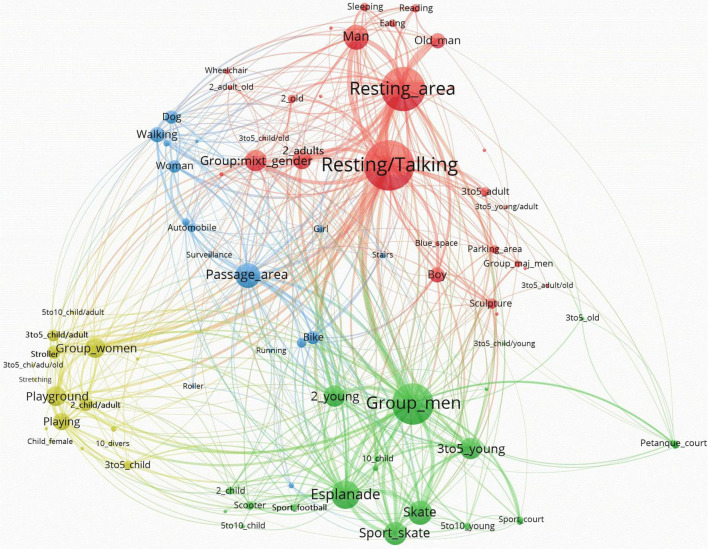
Three Chimneys Park’s network by VOSviewer.

In Three Chimneys’ Park, three major clusters and a second one are observed in the network (Figure 6). The former ones are clearly separate in the network layout, and from each of these patterns of use related to gender can be identified. The red cluster depicts two of the higher Weighted Degree nodes dealing with the *Resting_area* and the resting or talking activity (*Resting/Talking*) (see Table 3). Users in this cluster are mostly males (*Man*, *Old_man*, and *Boy*), in addition to mixed groups of gender (*Group_mixt*). *Sleeping* and *Reading* are the dominant activities appropriate for places like the *Resting_area*. The green cluster is characterized by groups of men (*Group*_*men*): young people (*2_young, 3to5_young, 5to10_young*) or children (*5to10_child, 10_child*) practicing skating (*Sport_skate*) in an *Esplanade*. The yellow cluster is mainly defined by groups of women (*Group*_wo*men*) with children (*Child_female, 3to5_child, 2_child/adult, 3to5_child/adult*, and *5to10_child/adult*), who are primarily located in *Playground_areas*, use *Strollers*, and are involved in *Playing* activities. The blue cluster is closely located to the red one. It is defined by the concurrence of single *Woman*, *Walking* alone, in *Passage_areas*. Remarkably, the red and blue clusters reflect the presence of single persons, while the green and yellow clusters represent groups of users.

**FIGURE 7:**
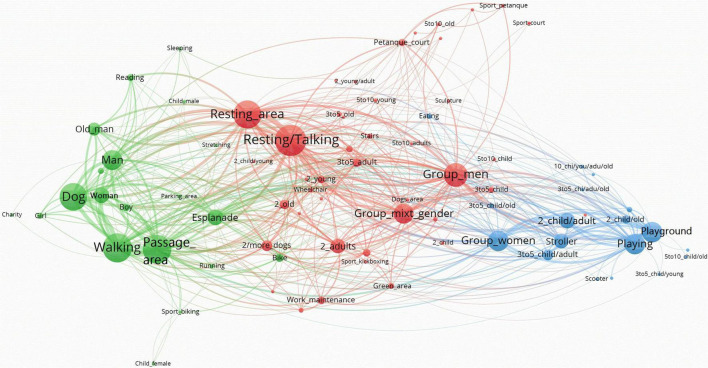
Pegaso Park’s network by VOSviewer.

Moreover, three well-defined clusters represent Pegaso Park’s network (Figure 7). The green cluster is related to single persons (*Man*, *Woman*, and *Old_man*), accompanied by a *Dog*, *Walking* in *Passage_areas* or in the *Esplanade*. These nodes have a large weight in the entire network (see Table 3). The red cluster includes nodes with a similar weight: groups of men (*Group*_*men*) talking or staying (*Resting/Talking*) in *Resting_areas*. In the extreme of the network, there are elder men (*5to10_old*) practicing petanque sport (*Sport_petanque*) in the *Petanque_court*. As in previous cases, the blue cluster is defined by groups of women (*Group*_wo*men*) with children (*2_child/adult, 3to5_child/adult, 5to10_child/adult, 2_child/old, 5to10_child*, and *3to5_child/young*) and *Strollers*, involved in *Playing* activities in the *Playground* area. The fact that Group_men (red cluster) and Group_women (blue cluster) are relatively close to each other in the network may suggest that, although clearly defined, the pattern of use belonging to the blue cluster, might not be as exclusive for women as in Three Chimneys and St. Pau Gardens.

## Discussion

Three urban public places in the city of Barcelona have been analyzed with the aim of recognizing patterns of use disclosing gender differences, which included St. Pau del Camp Gardens, Three Chimneys Park, and Pegaso Park. Based on an innovative combination of observational methodology and network analysis, several patterns of use of the public space were identified based on the gender profile of users, the occupancy of different functional areas, and the type of activity associated with them.

Firstly, general attributes of the networks such Network Density, Average Degree Index, or Centralization Network Index were found to be similar among the three networks. Technically speaking, all networks were similar, although they can vary in size (number of nodes and lines). Similarities were also detected when exploring the structure of the nodes within the networks. As a result, interesting regularities can be drawn.

People use to be in public places in the group, where male groups are generally predominant. Although the presence of lonely people in all areas was less frequent, in most cases there were male users. Whereas the occupation of the public space by women was preferably in playground areas, generally involving care activities concerned with children, men were found in resting and sports areas, either practicing sports or participating in leisure activities. Bearing in mind that the central activities identified in the observed places dealt with resting (by solitary people) or resting and talking (by groups of people), it is suggested that the main areas in which users were located have been characterized by a substantial presence of males. From the perspective of gender, another important activity such as walking was more balanced, although men used to be in the majority. These results are consistent with extant literature. As acknowledged previously (Franck and Paxson, 1989; Bondi and Rose, 2003; Neaga, 2014), there is a general tendency to relate certain activities and areas of use of the analyzed public spaces to the type of genre. Accordingly, women use to be associated with care activities, especially with children, and men with resting, sports, and leisure activities. Indeed, traditional patriarchal roles seem to contribute to the informal segregation of the public space yet (Blackstone, 2003). In spite of the changing times, as Koen and Durrheim (2010) noted about racial segregation, deeply inoculated social structures still appear informally when examining urban place dynamics. In line with Cao and Kang (2019), we showed that both men and women use to be in groups rather than alone; based on this, their activities and patterns clearly differ. Solitary people (predominantly men) tend to be in resting areas or walking in passage areas, often accompanied by dogs. Encounters of both groups of men and groups of women use to take place in resting areas to talk as well. However, unlike single persons, they occupied playgrounds (mostly women) and leisure areas such as sports courts, green areas, or blue spaces (mostly men). These gender differences are consistent with inequalities in the time spent by men on leisure activities (Codina and Pestana, 2019). According to these scholars, men spend more leisure time than women, while women have more positive leisure experiences. In this sense, in his analysis of Nordic cultures, Thrane (2000) claimed that during weekdays Danish, Swedish, and Norwegian women have less leisure time than their male partners, and these findings could be interpreted as in support of women’s double day effect.

Furthermore, differences in the design of the three urban sites can explain specific effects. For instance, the big esplanade in the Three Chimneys Park was found to be an attractive space for skaters. Skating is a different and a contemporary sports activity that makes this park unique compared to the other sites. However, most young men practiced it, with minimal participation of young women. This situation suggests that gender marginalization not only occurs in adults but also in young people, even when referring to a relatively new activity. The effect in skateboarding practice has been noted even in countries associated with high levels of gender equality such as Sweden (Bäckström, 2013). Once again, this supports the view about preestablished patriarchal roles regarding the informal segregation of certain activities.

A different pattern of use was observed in Pegaso Park. Due to its large extension and the variety of urban elements it contains (e.g., green areas, water areas, a long esplanade, an extended playground, and a petanque court) patterns according to gender were less intense. For example, the presence of men and women in areas concerned with activities such as resting, talking, walking, or leisure were more balanced than in the other sites. Compared to the other two cases, it is possible that the large variety of spaces and activities contributed to creating an intertwined network characterized by a complex use of the space (Park, 2020). For instance, Pegaso Park is the site that accommodates the largest number of sports activities. This complexity may have positively affected the fair appropriation and use of the public space. According to Dougherty (2006), an urban design that promotes a large variety of uses will promote psycho-environmental processes such as place attachment or spatial identity. This is because “identity and use are inevitably linked: much of a space’s identity depends on the uses that take place there and whether or not the space meets the needs of its users. In the same way, a space will not be used unless people can identify with it and feel a connection to it.” (op.cit, pp.II). Of course, such processes include gender identity: when variety is scarce, gender identarian connection is more restricted, and a specific gender occupation of the space can be prominent.

Similarly, with Three Chimneys Park, St. Pau Gardens’ network represented three clearly differentiating patterns related to single persons, groups of men, and groups of women, respectively. Conversely, in the latter, the activities and functional areas identified were more restricted than in the former: men (alone or in group) monopolize the resting area, while women in group were located in the playground. These findings suggest the existence of a kind of socio-spatial boundary that can be explained in terms of violence prevention against women. In this line, in a study about six public places in Barcelona, Pérez-Tejera et al. (in press) showed that women perceive these two public places as highly unsafe, St. Pau Gardens in particular. Such a perception can explain why women restrict their presence in public realms such as playgrounds, where they stay in the group.

To sum, the results confirmed that, despite the progressive consolidation of feminist urbanism as an influential line of thinking in urban planning and design, patterns of use in the public realm derived from traditional gender roles remained clearly recognizable. Therefore, from a gender perspective, a contribution of the study is that it informed about the main factors of the public space that reconfirmed the existence of such traditional patriarchal roles in our society – specifically the Catalonian society, as well as the importance of analyzing the public space as a key scenario where social aspects are explicitly exposed.

From a methodological perspective, processing observational data with network analysis techniques was shown to be relevant and adequate to tackle the intricacies of urban place analysis. The main advantage of this approach was that it allowed the collecting of large amounts of observational data straightforwardly. In comparison to more classical systems and approaches, network analysis enabled us to recognize and understand complex systems composed of numerous variables and relationships in a rather intuitive manner, which makes it a convenient instrument for architects and urban designers.

Therefore, implications for urban planning, urban design, and architecture can be suggested. From the perspective of these disciplines, public spaces must be considered key physical and functional components of the urban structure (Cudny and Appelblad, 2019). The urban public space is a never finished outcome of permanent change and adaptation. Through a proper comprehension of the urban phenomenon, designers and urban planners can positively influence the vitality of the public space. To this aim, they must be aware of how design affects the way people use and appropriate public spaces, and what activities they carry out there (Pasalar and Dewey Hallowell, 2019).

In the present study, we showed that the network analysis approach enabled us to easily and efficiently represent, analyze, and learn about the dynamics of the three case studies. In particular, it allowed us to gain a deep insight into the different patterns of space occupation and use in the different public spaces in terms of gender. Based on these, we propose that the network analysis tool can be integrated from the earlier stages of the urban planning and urban design process. In the first stage, this methodology should be used to collect objective complex data directly from an urban space under consideration through rigorous observation techniques. Then, this information can be visually represented and analyzed to identify the main patterns of use and to make a diagnosis about the suitability of the physical and functional features of the space. Eventually, the analysis of a large sample of public spaces will provide evidence about the existence of repetitive patterns (e.g., the profile of the user, type of space, and type of use or function), which may help understand and predict the extent to which the design of an urban realm may satisfy the users’ needs and requirements. Finally, the outcome of the network analysis tool will be critical to supporting future design interventions in the urban space. Urban design is a field that often deals with wicked problems and situations (Simon, 1984), which due to their complex and ambiguous nature are difficult to perceive, understand, and tackle. During the urban design process, designers and urban planners need to make assumptions and define initial requirements and goals, as well as clarify intentions and design ideas. They then construct problem situations that will lead to the development of design solutions (Cross, 2011). It is at this stage that the data collected and processed using network analysis can be integrated to help designers and planners define design priorities and frame design situations. Frames are critical structures of belief, perception, and appreciation (Schon, 1995). Accordingly, framing defines the boundaries of a design and guides subsequent logical design decisions (Dorst, 2015). Due to its processing and visual representation power of complex information, the network analysis approach can play a fundamental role in supporting the framing and decision-making process of designers and urban planners at this stage of the process.

There are several limitations to this study. Among them, is the inability to access qualitative relevant information. Although non-participant observation is a convenient technique to identify human behaviors in the public realm, it is not suitable to gain access to the users’ views about their reasons, preferences, and limitations for the use of the space. Accordingly, the use of mixed methods seems to be promising for future research. The use of Network Analysis in these environmental contexts is very incipient yet. Not much is known about the interpretation of centrality (referred to as nodes) and centralization (referred to as networks) indexes applied to urban life. In this study, we used some of these indexes, but their ability to inform about the social dynamics of the public places has yet to be explored. Finally, this work should be seen as a first step in studying patterns of use in the public space from an intersectional perspective. Although a major focus was set on gender analysis, we are aware of the importance of considering other identity adscriptions such as age, social status, or ethnicity, which is a combined analysis may contribute to gaining deeper insight into the use of the public space. Indeed, intersectionality is a key concept to be considered in research to come dealing with the social and urban design quality of public places in larger detail.

## Data Availability Statement

The raw data supporting the conclusions of this article will be made available by the authors, without undue reservation.

## Ethics Statement

Ethical review and approval was not required for the study on human participants in accordance with the local legislation and institutional requirements. Written informed consent for participation was not required for this study in accordance with the national legislation and the institutional requirements.

## Author Contributions

SV has collected and analyzed the data, collaborated in the discussion of the results, and wrote the first version of the manuscript. HC has participated in the theoretical framework of the study, collaborated in the discussion of the results, and wrote and corrected the final version of the manuscript. Both authors contributed to the article and approved the submitted version.

## Conflict of Interest

The authors declare that the research was conducted in the absence of any commercial or financial relationships that could be construed as a potential conflict of interest.

## Publisher’s Note

All claims expressed in this article are solely those of the authors and do not necessarily represent those of their affiliated organizations, or those of the publisher, the editors and the reviewers. Any product that may be evaluated in this article, or claim that may be made by its manufacturer, is not guaranteed or endorsed by the publisher.
